# Differences Between Right and Left Colon Cancer in Beira Interior

**DOI:** 10.7759/cureus.37500

**Published:** 2023-04-12

**Authors:** Catarina Gonçalves, Liliana Duarte, João José C Alves

**Affiliations:** 1 Internal Medicine, Hospital de Santa Maria, Centro Hospitalar Universitário Lisboa Norte, Lisboa, PRT; 2 Esophagogastric Surgery, Centro Hospitalar Tondela-Viseu, Viseu, PRT; 3 General Surgery, Centro Hospitalar Universitário Cova da Beira, Covilhã, PRT

**Keywords:** geographic factors, cancer epidemiology, right colon, left colon, colorectal cancer

## Abstract

Introduction

Colorectal cancer (CRC) is the second most common cancer in Portugal and worldwide, with a high mortality rate, especially in more advanced stages. In recent decades, there has been a growing interest in the distinction between right colorectal carcinoma (RCC) and left colorectal carcinoma (LCC) due to the different presentation, treatment, and prognosis. Studies show that RCC and LCC have different clinical and biological characteristics, being considered two distinct entities.

Material and methods

This cross-sectional, descriptive, and comparative retrospective study included data collection at the three hospitals of Beira Interior - Centro Hospitalar Cova de Beira, Hospital Amato Lusitano, and Hospital Sousa Martins - over a 6-year period.

Results

The proportion of RCC cases was higher. The proportion of women was higher in the RCC group compared to the LCC (46.2%, 121/262 vs. 39%, 76/195). Anemia was statistically higher in the RCC group (p <0.01). On the other hand, intestinal occlusion tends to appear in patients with LCC (p <0.001). The most frequent surgery was elective. The proportion of emergency surgery was higher in the LCC group (LCC vs RCC: 27.2% vs 18.3%; p = 0.03).

Discussion and conclusion

In both the RCC and LCC groups, the male sex is the most frequently observed in Beira Interior and in Portugal, opposite from the world population, in which the female sex predominates in patients with RCC. The RCC presents alterations in bowel habits more often (p> 0.05). On the other hand, anemia is more common in RCC and intestinal occlusion in LCC, following the current literature. Conducting targeted studies and optimizing the screening and treatment processes are key to reducing mortality associated with CRC.

## Introduction

Colorectal cancer (CRC) is the second most frequent in Portugal and worldwide, with a high mortality rate [1], especially in the more advanced stages. In recent decades, there has been a growing interest in distinguishing between right colorectal carcinoma (RCC) and left colorectal carcinoma (LCC) due to the different presentation, treatment, and prognosis. Studies have shown that right colon carcinoma (RCC) and left colon carcinoma (LCC) have different clinical and biological characteristics, and are currently considered two distinct entities. The basis of the observed differences could be explained by the different embryonic origins.

The right colon is composed of the cecum, ascending colon, hepatic angle, and proximal transverse colon. In turn, the left colon comprises the distal transverse colon, the splenic angle, the descending colon. and the sigmoid colon. The rectum has the same embryological origin as the entire left colon, that is, in the hindgut.

There are anatomical and physiological differences between the two that may justify the distinction, from molecular pathways to others such as gender, age at presentation, TNM stage at diagnosis, histological type, and clinical presentation [2]. There is a significantly higher proportion of female patients with RCC compared to the distribution of LCC [3].

Patients with RCC present, at the time of diagnosis, a more advanced TNM stage than patients with LCC [4,5]. This fact can be attributed to early symptomatology (microcytic anemia, weight loss) present in the RCC, which leads to the search for advanced medical assistance in these cases, unlike cases of LCC that usually have later symptoms (lower gastrointestinal tract bleeding and changes in bowel habits).

The more advanced stage in RCC is associated with an increase in tumor size, increased tumor vascularization, and a marked invasive character, while polypoid and annular tumors arise in RCC, usually associated with clinical conditions of intestinal occlusion [6-8].

In RCC, mucinous, signet-ring and undifferentiated cell carcinomas appear more frequently. On the other hand, SCC is almost always diagnosed with differentiated adenocarcinoma [9]. Studies support the hypothesis that the physiological basis of these differences lies in complex genetic mechanisms and epigenetic changes as a result of the combination of hereditary and environmental factors.

In Portugal, RCC represents 17.6% of the population's cancer cases, being the second most incident and also the second with the highest associated mortality rate. The incidence in women is slightly lower (16.2%) than in men (18.8%) [10].

There are some studies on the pathology in some specific Portuguese regions, such as the Autonomous Region of Madeira [11].

## Materials and methods

A retrospective cross-sectional, descriptive, and comparative study was carried out with data collection in the three hospitals of Beira Interior - Centro Hospitalar Cova de Beira, Hospital Amato Lusitano, and Hospital Sousa Martins -, corresponding over 6 years.

The incidence of colon carcinoma was analyzed according to location, gender, age groups concerning gender, distribution in histological types, and type of surgery.

Data were collected from 666 cases, including 457 patients with colon cancer admitted to a general surgery service between 2010 and 2015. Of the 666 cases, 209 cases were excluded, corresponding to patients under 18 years of age, patients with concomitant carcinoma of the rectum or appendix, patients with synchronous colorectal cancer, patients with relapsed colorectal cancer, patients who could not be classified as RCC or LCC. and with dossiers with insufficient data.

This study is composed of qualitative and quantitative variables. A descriptive analysis was performed to describe and summarize the data. For the descriptive statistical analysis, the Statistical Software SPSS® version 24.0 for Microsoft Windows® was used.

Some statistical inference techniques were used, namely the chi-square test (X^2^) and odds ratio (O.R.), as this is a retrospective study. Quantitative variables were studied using Student's t-test and Mann-Whitney U test. In this study, p<0.05 was considered statistically significant.

## Results

In this study, the proportion of RCC cases was higher. The proportion of women was higher in the RCC than in the LCC (46.2%, 121/262 vs. 39%, 76/195). The age of patients with RCC was significantly higher than that of patients with LCC (72.6 ± 10.8 vs. 70.2 ± 12.7; p <0.01), emphasizing a greater proportion of LCC cases in the earlier age group.

In both groups, the increase in the number of cases follows the increase in age, and the difference between the distribution of the two groups - RCC and LCC - is not significant (Table 1).

**Table 1:** Distribution of patients in the various age groups at the RCC and LCC

Group ages	RCC	LCC
29-40	0.20%	1.10%
41-65	14%	11.20%
66-75	16.80%	13.80%
76-95	26.30%	16.60%

Regarding the proportion of genders in the different age groups, the differences within the groups explained in Table 1 were analyzed (Table 2).

**Table 2:** Comparison of incidences by age groups and sex

Group ages	RCC		LCC	
	WOMEN	MEN	WOMEN	MEN
[29;40]	0.8%	0.0%	2.6%	0.0%
[41;65]	18.2%	29.8%	9.2%	16.9%
[66;75]	27.3%	31.2%	11.3%	21%
[76:95]	53.7%	39.0%	15.9%	39%

The youngest age group in both groups was composed solely of women. In the next two ranges, both groups had a higher proportion of male patients, differing in the last range, in which the RCC group had a greater number of female patients, unlike the LCC group. The groups showed differences in the proportions of gender in the distribution by age groups (p <0.05).

In the sample, the most common presenting symptoms of the RCC were anemia, change in bowel habits, asthenia, anorexia, and screening. In LCC, the most common presenting symptoms were lower gastrointestinal bleeding, intestinal occlusion, abdominal pain, and others.

The proportion of patients with anemia was statistically higher in the RCC (p < 0.01). On the other hand, intestinal occlusion tends to appear in patients with LCC (p < 0.001).

The most frequent postoperative TNM stages are II in RCC and III in LCC. Descriptively, the most frequent stage in RCC is IIA, while in LCC it is IIIB. However, the difference between them didn't present statistical significance.

The most frequent histological type in both groups - RCC and LCC - is adenocarcinoma (96.6% vs. 99%). Mucinous tumor with signet-ring cells represents 0.8% and 1% of RCC and LCC tumors, respectively. In the LCC group, there were no cases of tumors with other histological types. There was no statistical significance (Table 3).

**Table 3:** Comparison of distribution by histological types in RCC and LCC

	ADENOCARCINOMA	SIGNET RING CELL CARCINOMA	Others
RCC	96.60%	0.80%	2.70%
LCC	99%	1.00%	

The most frequent type of surgery was elective, with a higher proportion of cases in both groups. However, in LCC, the proportion of emergency surgeries was higher (LCC vs RCC: 27.2% vs 18.3%; p = 0.03).

## Discussion

CRC is one of the most common cancers worldwide. In 2018, death associated with CRC accounted for 5.8% of all deaths [10]. Currently, it is known that right colon carcinoma and left colon carcinoma exhibit differences according to gender, age, and geographic region, and they should be considered two different entities [12]. There are many studies on these differences: the pathophysiology and the genetic pathways involved, age and symptomatology of presentation, stage of presentation, prognosis, chemotherapy scheme used, premalignant lesions, and risk factors [3-5,8,9,13-20].

The distinction between RCC and LCC has a great impact on monitoring, treatment, and prognosis [20]. In the last decade, there has been an increase in the incidence of RCC compared to LCC, which has remained stable or has decreased [21-22], according to the prediction presented in some studies [2,15].

In recent epidemiological studies, the prevalence of LCC is evident, although the incidence of RCC has been increasing in recent years [2]. The prevalence of RCC in age groups up to 40 years is considerably high [3] and higher in women when comparing the two genders [3]. The data from the sample under study follow the international trend that RCC is more common than LCC, showing a reversal of the trend documented in Portugal and previously in the world [2,12].

Some factors may have influenced this reversal, such as the implementation of screening through the Fecal Occult Blood Test (FOBT) [6,15,17]. There is evidence that flexible sigmoidoscopy decreases the incidence and mortality associated with LCC, taking into account the difficulty of performing a total colonoscopy compared to sigmoidoscopy and the difference in associated costs. Colonoscopy may not be 100% effective in visualizing the structures of the right colon, being better in visualizing the other structures [17], contributing to this reversal by removing adenomatous polyps present mostly in the LCC [13].

The distribution by gender does not differ from international trends [3]. However, both in the RCC and in the LCC, males are the most frequent in Beira Interior and Portugal, which differs from the world population, where RCC is more frequent in females [3,8].

International data demonstrate a greater number of cases of RCC in younger age groups, compared to LCC. The subtle symptomatology of RCC is pointed out as responsible for underdiagnoses of the pathology in these age groups [23].

The studied sample differs from international trends in earlier age groups, showing a higher proportion of LCC cases (29-40) in comparison [3]. RCC is more frequently presented by changes in bowel habits (p >0.05). On the other hand, anemia is more common in RCC and intestinal obstruction in LCC, in line with the current literature [15,19].

The international literature exposes stage II as the most frequent presentation [21], the number of cases of RCC in stage I is small, and in stage III very high [12]. In this sample, the number of cases of RCC diagnosed in stage II is the most representative, with stage III being the most frequent in LCC.

## Conclusions

In Portugal, RCC represents 17.6% of diagnosed cancers, which has increased in the last 30 years. This increase is strongly associated with changes in lifestyle, namely exposure to carcinogens, high calorie and fat intake, as well as tobacco consumption. Despite being a widely studied area, there is still much to discover. Carrying out population studies correlated with various constraints like the age pyramid, diet, and geographic area is important to obtain a greater understanding of RCC. Currently, in Portugal, colon cancer is screened nationwide. Unfortunately, in 2012, only 9.3% of the Portuguese population was covered by screening. In 2016, there was an attempt to increase the coverage of the Portuguese National Screening, increasing the number of individuals screened to 31%, with new measures designed for greater population coverage. The computerization of the National Health Service, a project started in 2017 by Shared Services of the Ministry of Health, has also contributed to the implementation of screening programs, allowing for the centralization and standardization of processes. Conducting targeted studies and optimizing screening and treatment processes are key to reducing CRC-associated mortality.
